# Pirfenidone modifies hepatic miRNAs expression in a model of MAFLD/NASH

**DOI:** 10.1038/s41598-021-91187-2

**Published:** 2021-06-03

**Authors:** Rebeca Escutia-Gutiérrez, J. Samael Rodríguez-Sanabria, C. Alejandra Monraz-Méndez, Jesús García-Bañuelos, Arturo Santos-García, Ana Sandoval-Rodríguez, Juan Armendáriz-Borunda

**Affiliations:** 1 Department of Molecular Biology and Genomics, Institute for Molecular Biology in Medicine and Gene Therapy, Health Sciences University Center, University of Guadalajara, Guadalajara, Jalisco Mexico; 2 Tecnologico de Monterrey, School of Medicine and Health Sciences, Zapopan, Jalisco Mexico; 3 Institute for Molecular Biology and Gene Therapy, CUCS, University of Guadalajara, 950 Sierra Mojada, 44340 Guadalajara, Mexico

**Keywords:** Gastroenterology, Hepatology, Liver diseases, Non-alcoholic steatohepatitis

## Abstract

miRNAs are involved in the development of metabolic associated fatty liver disease (MAFLD) and nonalcoholic steatohepatitis (NASH). We aimed to evaluate modifications by prolonged-release pirfenidone (PR-PFD) on key hepatic miRNAs expression in a MAFLD/NASH model. First, male C57BL/6J mice were randomly assigned into groups and fed with conventional diet (CVD) or high fat and carbohydrate diet (HFD) for 16 weeks. At the end of the eighth week, HFD mice were divided in two and only one half was treated with 300 mg/kg/day of PR-PFD mixed with food. Hepatic expression of miRNAs and target genes that participate in inflammation and lipid metabolism was determined by qRT-PCR and transcriptome by microarrays. Increased hepatic expression of miR-21a-5p, miR-34a-5p, miR-122-5p and miR-103-3p in MAFLD/NASH animals was reduced with PR-PFD. Transcriptome analysis showed that 52 genes involved in lipid and collagen biosynthesis and inflammatory response were downregulated in PR-PFD group. The expression of *Il1b*, *Tnfa*, *Il6*, *Tgfb1*, *Col1a1*, and *Srebf1* were decreased in PR-PFD treated animals. MAFLD/NASH animals compared to CVD group showed modifications in gene metabolic pathways implicated in lipid metabolic process, inflammatory response and insulin resistance; PR-PFD reversed these modifications.

## Introduction

Metabolic associated fatty liver disease (MAFLD), previously known as non-alcoholic fatty liver disease is currently the most common cause of chronic liver disease worldwide^1,2^. MAFLD is characterized by hepatic steatosis accompanied by one of three features: overweight or obesity, type 2 diabetes mellitus (T2DM), or either lean or normal weight with evidence of metabolic dysregulation^3^. MAFLD prevalence is rising due to the global obesity epidemic and high prevalence of type II diabetes mellitus^4,5^. It is estimated that up to a third of the world population will present the disease in a nearby future^6,7^. Population studies have estimated a prevalence of 17–22% in asymptomatic patients^8,9^. The progressive form of MALFD condition; nonalcoholic steatohepatitis (NASH), is defined by liver cell damage, lobular inflammatory infiltrate, and a variable degree of fibrosis^10^. Several microRNAs (miRNAs) are involved in MAFLD progression and NASH pathology. miRNAs are highly conserved non-coding RNAs that epigenetically regulate gene expression at post-transcriptional level. miRNAs involved in lipid synthesis, catabolism of fatty acids and glucose and inflammation, are known to be dysregulated in MAFLD/NASH^11,12^; including miR-122-5p, miR-34a-5p, miR-21a-5p and miR-103-3p^13–16^. miR-122-5p is the most expressed miRNA in adult human liver (approximately 70% of total miRNAs) and its key role in total serum cholesterol regulation and liver lipids metabolism has been clearly demonstrated^17,18^. miR-34a-5p is involved in lipid metabolism, apoptosis, cell cycle control, and studies in mice have shown how overexpression of miR-34a-5p increases the level of liver triglycerides^19,20^. Likewise, it has been demonstrated that miR-103-3p regulates insulin sensitivity and glucose homeostasis^21,22^; while miR-21a-5p may be involved at different stages of MAFLD progression, in buildup of lipids, appearance of steatosis in hepatocytes and/or inflammation at early stages and fibrosis at a later stage of the disease^23,24^.

Several drugs are currently being investigated for the treatment of NASH, notwithstanding, there are no current FDA approved therapies for treating NASH. Given the vast complexity of NASH where lipid metabolism, insulin resistance, oxidative stress, inflammation and fibrosis pathways are involved, a drug with multiple biological targets ought to be required to stimulate beneficial effects, if any^25,26^. Pirfenidone (PFD) is a pyridone-derived drug with extensive antifibrotic, anti-inflammatory and antioxidant effects which modulates a number of cytokines, i.e.,* Tgfb1, Il1, Il4, Il6, Il8, Il13, IFNG *and* Tnfa*, among others. Furthermore, PFD reduces expression of intracellular adhesion molecule 1 (*Icam-1*) and improves anti-inflammatory cytokines expression, such as *Il10*^27,28^. In this work we used prolonged-release pirfenidone (PR-PFD) with an improved bioavailability compared to standard pirfenidone and a half-life of ~ 8 h^29^. Thus, the aim of this study was to evaluate modifications by PR-PFD on key hepatic miRNAs expression in a MAFLD/NASH model.

## Methods

### Drug

Prolonged-release pirfenidone (PR-PFD) was donated by Cell Pharma S.A. de C.V. (Cuernavaca, Mexico). Chemical reagents used were reactive grade.

### Animals

CUCS Research Committee at the University of Guadalajara approved this study (protocol number: CI-00518). The study was carried out in compliance with the ARRIVE guidelines. All mouse assays were in compliance with the Animal Research Reporting in Vivo Experiments guidelines and accomplished according to guidelines for the care and use of laboratory animals published by the US National Institutes of Health (NIH, publication No. 85-23, revised 1996). Four-week-old male C57BL/6J mice (n = 15), weighing 18–22 g, were obtained from Cinvestav Animal Facility (Mexico City). After a week of acclimatization, animals were divided into groups; fed with conventional diet (CVD) with 18% lipids (2018S.15-Envigo) and plain water or fed with high fat/high carbohydrate diet (HFD) with 60% fat nutriment (TD.06414-Envigo) and 42gr sugar/L in drinking water (55% fructose and 45% sucrose) for 16 weeks. From ninth week to the end of the protocol, a subgroup of five HFD mice were treated with prolonged release pirfenidone (PR-PFD) mixed with food (300 mg/kg/day). Mice were maintained with 12-h light/dark cycles, ad libitum food and water; and constant temperature and humidity. Animals were weighed weekly to control weight gain.

### Sacrifice, clinical and biochemical parameters

Animals were sacrificed after 4 h of fasting using tiletamine/zolazepam (15 mg/kg/bw, Zoletil 50, Virbac) at the end of the 16th week of model induction. Liver and whole blood were collected. The weight of epididymal fat and liver were determined. Blood was centrifuged to obtain serum where lipid profile (triglycerides, total cholesterol and VLDL) and liver enzymes (AST, ALT) were determined using VITROS DT60II dry chemistry analyzer (Johnson & Johnson).

### Insulin tolerance test and determination of glucose levels and HOMA

Fasting blood glucose was measured weekly during the study in mouse-tail vein using a clinical glucometer. The Insulin Tolerance Test (ITT) was determined at eighth and sixteenth week. After 4 h-fasting, 0.025 U/mouse of short-acting human recombinant insulin was intraperitoneally administrated and glucose levels were measured at 0, 30, 60 and 90 min after insulin injection. The area under the curve was calculated using the trapezoidal method^30^. Insulin was measured in serum by multiplex detection immunoassay. Insulin resistance (IR) was calculated using the homeostasis model assessment (HOMA)-IR index with the following formula [fasting serum glucose (mg/dL) × fasting serum insulin (μIU/mL)/405]^31^.

### Histological analysis of liver

Samples from the three main liver lobes were fixed in 4% paraformaldehyde (0.1 M PBS, pH 7.4). Paraffin embedded tissues were cut into 5 µm sections. Hematoxylin–eosin, Masson's trichrome and Sirius red staining were performed. Masson's and H&E were analyzed by two independent pathologists to quantify inflammation, microvesicular steatosis and macrovesicular steatosis. Microvesicular steatosis was defined by the presence of numerous small lipid vacuoles that do not displace the nucleus; while macrovesicular steatosis was defined by a large lipid vacuole that displaces the nucleus. In addition, periportal and centrilobulillar fibrosis were examined by scoring fibrotic bridges. To evaluate hepatic stellate cells activation, immunoreactivity against alpha-smooth muscle actin (Cell Signaling Technology Inc., Beverly MA, USA) and anti-GFAP (Glial Fibrillary Acidic Protein) (Biocare Medical, Pacheco, CA, USA) was performed using a 1:50 and 1:100 ab dilution respectively. To evaluate inflammatory infiltration in the liver immunoreactivity against CD68 (Biocare Medical, Pacheco, CA, USA) was carried out using a 1:100 ab dilution.

Collagen, alpha-smooth muscle actin, GFAP and CD68 staining were evaluated using Image-Pro software in 30 photographs at 20 × microscopic field magnification (Media Cybernetics, Inc., Bethesda MD, USA).

### RT-qPCR

RNA isolation from liver was performed according to Chomczynski and Sacchi modified method^32^. RNA quantity and quality were determined with NanoDrop equipment (Thermo Scientific, USA). 2 µg of total RNA were used for retrotranscription with 240 ng Oligo dT, 0.5 mM dNTPs mix, 10 mM DTT, 2 U of RNAse inhibitor and 200 U M-MLV (Invitrogen, Carlsbad, CA). qPCR reactions were performed on the LightCycler 96 Instrument (Roche Molecular Systems, Pleasanton, CA, USA). All data were run in triplicate, normalized using GAPDH as housekeeping gene and data analysis was performed using the 2^−Δct^ method^33^. Information about the probes used is shown in Supplemental Table 1.

### miRNAs extraction and expression

Representative liver tissue was stored in RNAlater stabilization solution (Ambion, Austin, TX, USA) at − 80 °C. miRNAs were extracted using the miRVana miRNA isolation kit (Thermo Fisher Scientific, Waltham, MA, USA), according to manufacturer's instructions. The integrity and amount of the RNA fraction highly enriched in sRNA species ≤ 200 nt was determined using a Nanodrop ND-2000 spectrophotometer (Thermo Fisher Scientific, Waltham, MA, USA). RNA purity was assessed from the ratio of A260/A280; where 1.8–2.1 is expected^34^. Ten ng of sRNAs were used to performed retrotranscription using the Advanced miRNA cDNA synthesis kit (Thermo Fisher Scientific, Waltham, MA, USA), following the manufacturer's instructions. Reverse transcription products were diluted to 50 µL, and 2.5 µL of the diluted sample were used for qPCR reactions, with a total volume of 10 µL/Rx and 1X Thermo specific TaqMan probes were used for qPCR for mmu-miR-34a-5p, mmu-miR-122-5p, mmu-miR-21a-5p and mmu-miR-103-3p, in a LigthCycler 96 equipment (Roche Molecular Systems, Pleasanton, CA, USA). qPCR was performed as follows: 1 cycle at 95 °C for 20 s, 40–60 cycles 95 °C for 1 s followed by 60 °C for 20 s. Probes catalog number are shown in Supplemental Table 1. Gene expression was normalized against mmu-miR-16-5p. Analysis was performed using the 2^−ΔCT^ method^35^.

### Microarrays

Total RNA was extracted from 20 mg of liver tissue using Trizol reagent (Invitrogen, Carlsbad, CA, USA). Abundance and ratio between 28 and 18S rRNA were monitored both by agarose gel electrophoresis and NanoDrop ND-2000 spectrophotometer lectures (Thermo Fisher Scientific, Waltham, MA, USA). Total RNA pooled from 5 animals per group was used throughout the experiment. Reverse transcription and RNA sample labeling was made according to Microarray Facility UNAM protocol (http://microarrays.ifc.unam.mx)^36^. Double-channel microarrays for *Mus musculus* genome were used and 22,000 genes were hybridized. Image quantification was analyzed with genArise Microarray Analysis tool version 2.0 with adjusted p values of < 0.05 and Z score values of > 1.5 and < 1.5 were considered to be significant. To provide functional interpretation of enlisted genes, **D**atabase for **A**nnotation, **V**isualization and **I**ntegrated **D**iscovery (**DAVID**) v6.8 was employed to visualized the cell pathways and processes involved in non-alcoholic steatohepatitis and modifications induced by PR-PFD^37^.

### Statistical analysis

Values are expressed as mean ± SD, or as mean ± SEM. Groups were compared with one-way ANOVA followed by Tukey test for parametric data and Kruskal–Wallis or U de Mann–Whitney for non-parametric data. Values of p < 0.05 were considered statistically significant. Analysis was run with GraphPad Prism version 8.

## Results

### Pirfenidone improves clinical and biochemical parameters in MAFLD/NASH animal model

All animals gradually increased their weight during the experimental period. As seen in Table 1 after being fed a high-fat diet for 16 weeks, HFD mice showed a 2.8-fold increase in body weight; while PFD group gain a 1.96-fold increase in weigh compared to CVD group. CVD fed animals showed 27.98 g ± 0.47 of body weight. HFD mice treated with PR-PFD, diminished body weight 18.11% against HFD group (40.33 g ± 2.82 vs 49.25 g ± 2.36; p < 0.05). These data indicated that PFD-treated animals even when fed with HFD, ended with lesser weight gain and final body weight. No statistical differences in liver weight or liver/animal weight ratio were observed in any experimental group (Table 1). Epididymal fat pad showed a significant decrease in PFD group compared to animals in HFD group (1.79 g ± 0.16 vs 2.60 g ± 0.33; p < 0.05) (Table 1). Also, PFD group displayed a significant decrease in AST and ALT serum levels (p < 0.0001) versus HFD group (Table 1). Triglycerides, cholesterol and VLDL serum levels were higher in HFD group mice against PFD group (p < 0.0001) (Table 1). Animals treated with pirfenidone had a slight decrease in serum glucose compared to HFD group. However, mice receiving PFD treatment showed increased insulin sensitivity (AUC determinations), which was statistically significant at the end of week 16, (p < 0.05). Insulin levels were increased in HFD animals compared to CVD group (4326 ± 446.3 vs 2490 ± 321.6; p < 0.05) (Table 1). Insulin showed no statistical differences between HFD and Pirfenidone group. Insulin resistance was increased in HFD group, as assessed by HOMA-IR, yet an increase in this hormone sensitivity was observed in mice treated with PFD.

**Table 1:** Clinical, histological and biochemical parameters in animal groups.

Parameter	Group		
	CVD	HFD	PFD
**Body weight (g)**			
Initial	17.38 ± 0.31	20.60 ± 0.67	19.30 ± 0.46
Final	27.98 ± 0.47	49.25^a^ ± 2.36***	40.33^b^ ± 2.82*
Weight gain (g)	10.60 ± 0.51	29.54^a^ ± 1.84***	20.83^b^ ± 2.97*
Epididimal fat pad weight (g)	0.67 ± 0.11	2.60 ± 0.33	1.79^b^ ± 0.16*
Relative liver weight (g)	4.85 ± 0.34	4.7 ± 0.30	4.7 ± 0.30
Macrovesicular steatosis (%)	–	19.2 ± 1.1	8.0^b^ ± 1.8****
Microvesicular steatosis (%)	–	71.2 ± 1.7	67.7 ± 2.3
Inflammatory nodules number/total area	–	14.8 ± 2.1	6.60^b^ ± 0.96***
Fibrotic bridges number/total area	–	4.6 ± 1.9	0.6^b^ ± 0.2*
Periportal fibrosis (%)	–	72.5 ± 7.3	34.7^b^ ± 3.8**
Pericental fibrosis (%)	–	34.2 ± 3.3	20.5^b^ ± 3.1*
AST (U/L)	116.7 ± 6.7	225.0^a^ ± 8.6****	140.0^b^ ± 19.2****
ALT (U/L)	36.0 ± 2.3	91.0^a^ ± 1.7****	42.0^b^ ± 2.8 ***
Total colesterol (mg/dL)	78.0 ± 6.3	153.0^a^ ± 7.7*	120.0^b^ ± 6.9*
Triglycerides (mg/dL)	128.0 ± 8.0	212.0^a^ ± 12.7****	127.0^b^ ± 10.9****
VLDL (mg/dL)	30.0 ± 1.7	34.0 ± 2.0	25.0^b^ ± 2.3*
Serum glucose (mg/dL)	110.7 ± 6.93	207.0^a^ ± 14.21**	177.3 ± 5.48
AUC	9.65 ± 0.62	18.09 ± 1.69	11.73^b^ ± 0.35*
Insulin (μIU/mL)	2490 ± 321.6	4326^a^ ± 446.3*	4710^a^ ± 719.4*
HOMA-IR index^c^	1.0 ± 0.06	3.25^a^ ± 0.36**	2.7 ± 0.54

Data are expressed as mean ± SEM. *CVD* conventional diet, *HFD* high fat and high carbohydrate diet, *PFD* high fat diet + prolonged release pirfenidone. *VLDL* very low-density lipoproteins.

^a^Significance difference versus CVD group (*p < 0.05, ****p < 0.0001).

^b^Significance difference versus HFD group (*p < 0.05, **p < 0.01, ***p < 0.001, ****p < 0.0001).

^c^Fasting serum glucose (mg/dL) × fasting serum insulin (μIU/mL)/405.

### Pirfenidone modifies MAFLD/NASH-involved miRNAs expression while improving liver inflammation and steatosis

**Figure 1:**
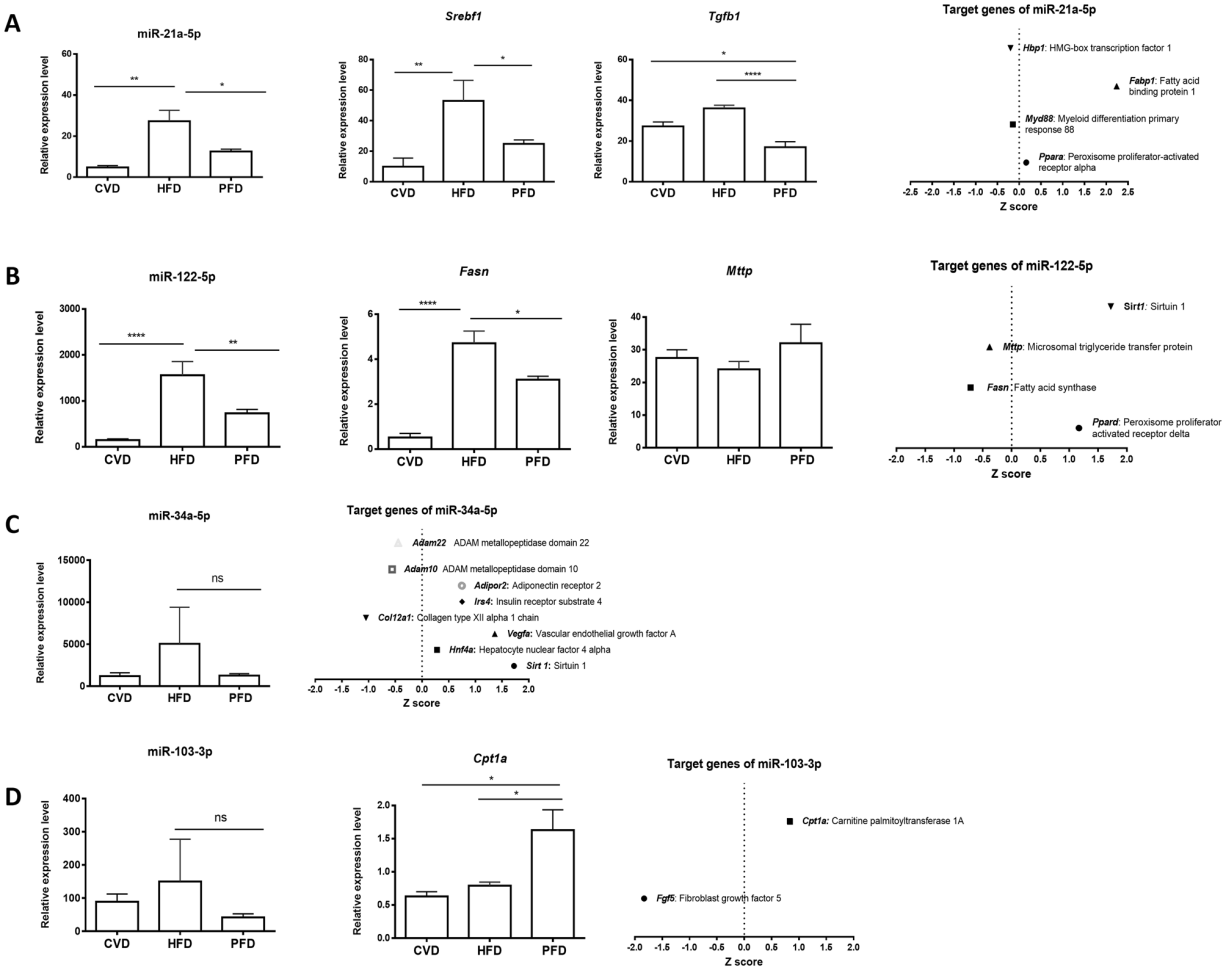
Hepatic expression of selected miRNAs involved in the pathogenesis of non-alcoholic steatohepatitis and their target genes. (**A**) miR-21a 5p, *Srebf1* and *Tgfb1* gene expression by qPCR. Final panel presents microarray analysis for several miR-21a 5p target genes. (**B**) Expression analysis of miR-122-5p and mRNA levels of *Fasn* and *Mttp*; and microarray of miR-122-5p target genes. (**C**) miR-34a-5p gene expression and microarray analysis of targeted genes. (**D**) Levels of miR-103-3p and *Cpt1a* by qPCR, and microarray analysis of miR-103-3p targeted genes. *CVD* conventional diet, *HFD* high fat and high carbohydrate diet, *PFD* high fat and high carbohydrate diet + prolonged-release pirfenidone. *p < 0.05, **p < 0.01, ****p < 0.0001.

Several miRNAs involved in MAFLD/NASH development where explored using RT-qPCR in liver tissue. Also, in order to correlate changes in miRNAs expression, some of their target genes were selected on miRbase and RT-qPCR was performed and/or were searched in the microarray database of PFD vs HFD chip. Liver levels of miR-21a-5p, a key regulator of lipid metabolism and hepatic fibrogenesis, were significantly higher (27.28 ± 5.27) in HFD mice compared to CVD mice (4.8 ± 0.85; p < 0.01). HFD mice receiving PFD treatment exhibited a significant lower expression (12.52 ± 1.15; p < 0.05) (Fig. 1A). *Srebf1* and *Tgfb1* mRNA hepatic expression were determined using PCR. *Srebf1* mRNA levels were lower in mice administered with PFD (24.82 ± 2.62; p < 0.05) versus HFD group (52.94 ± 13.51), while *Tgfb1* also revealed statistical negative difference (17.00 ± 2.67 vs 36.09 ± 1.53; p < 0.0001) (Fig. 1A). Additionally, data on microarray analysis showed upregulation in miR-21a-5p-target genes like *Fabp1* (2.24) and Ppara (0.16); while *Hbp1* and Myd88 showed downregulation (− 0.20 and − 0.15, respectively).

miR-122-5p levels are elevated under lipid buildup conditions, with quantities correlating with NASH scoring and severity. As shown in Fig. 1B, our results revealed that miR-122-5p was upregulated in hepatic tissue of HFD fed mice, and concurrent treatment with PFD decreased its liver expression (1559 ± 296.50 vs 730.80 ± 85.52; p < 0.01). Target genes for miR-122-5p like *Fasn*, *Mttp*, *Sirt1* and *Ppard* showed in microarray profile − 0.71, − 0.38, 1.72 and 1.16 expression values compared to HFD animals. While qPCR expression of *Fasn* was found to decrease (3.08 ± 0.15; p < 0.05) in PFD group against HFD group (4.70 ± 0.54). By qPCR, *Mttp* gene expression showed non-statistical difference between groups.

miR-34a-5p is usually expressed in the liver, modulating oxidative stress and lipid metabolism. Figure 1C shows its increased expression in HFD group compared to PFD administered animals (5060 ± 2844 vs 1264 ± 149.0; p = 0.12). Noteworthy, PFD group levels are similar to those obtained in CVD group (1208 ± 260.4). miR-103-3p has been shown to regulate insulin sensitivity and glucose homeostasis and is highly expressed in the liver of MAFLD patients. Data in Fig. 1D for miR-103-3p indicated an upregulation in HFD animals (150 ± 85.08) and a notable decrease in its expression after PFD treatment (42.14 ± 7.19; p = 0.25). In microarray assays, its target gene *Fgf5* was found downregulated (− 1.82); while *Cpt1a* was found upregulated (0.84) in PFD group. Concurrent with this finding, *Cpt1a* is upregulated in animals treated with PFD (1.630 ± 0.3047, p < 0.05) compared to the high fat diet group (0.792 ± 0.05).

### MAFLD/NASH-related parameters are decreased in liver tissue of animals after pirfenidone administration

**Figure 2:**
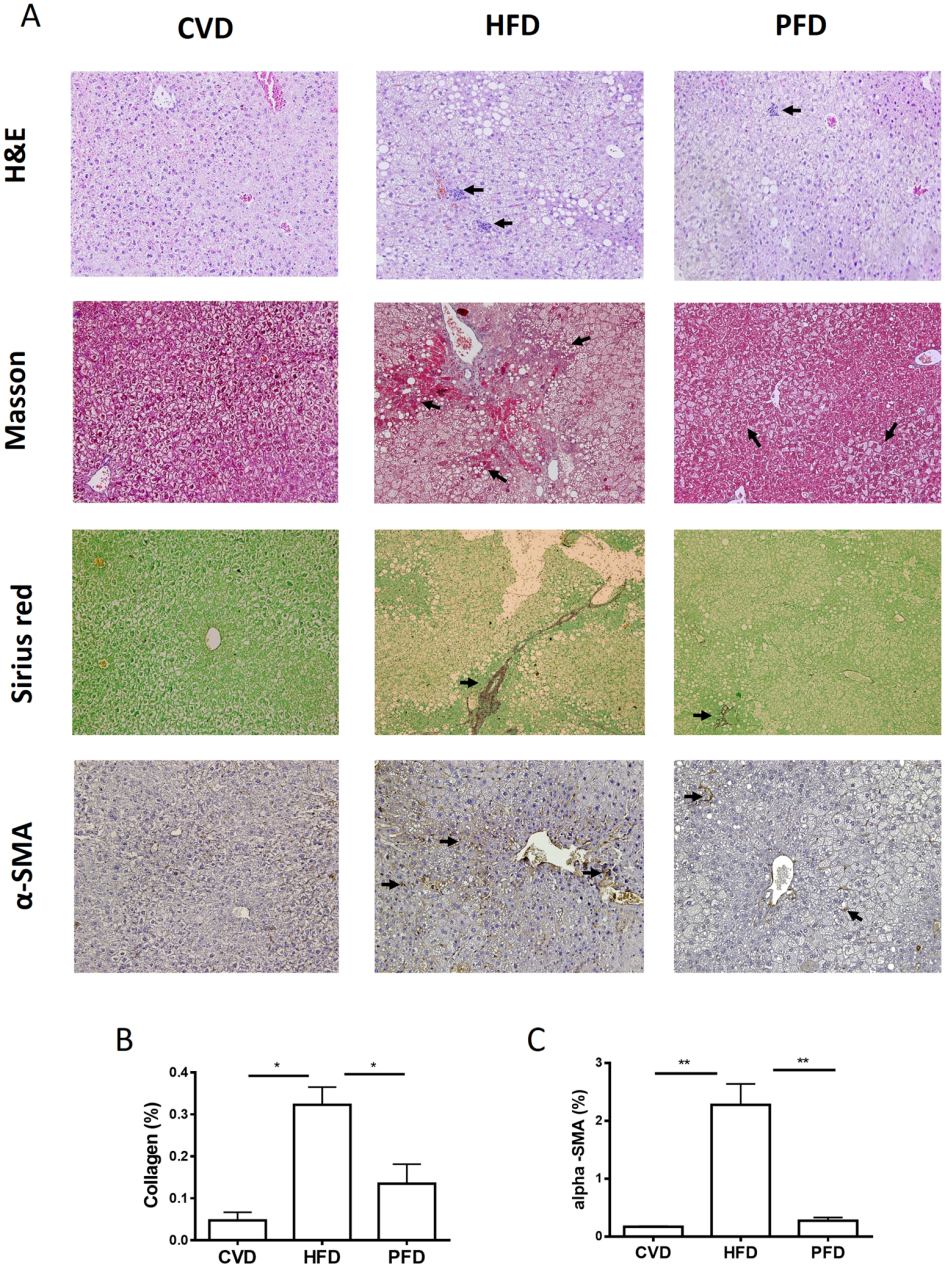
Histological assessment. (**A**) Microscopic photographs of liver sections. In animals with NASH, an intense inflammation is observed, constituted by neutrophils and mononuclear cells (arrows). Animals treated with PFD showed reduction of inflammatory nodules in H&E-staining. Masson staining showed initial fibrotic bridges and macrovesicular steatosis in animals fed with HFD. Fibrotic bridges and macrovesicular steatosis were diminished in animals treated with PFD. IHC for alpha-SMA revealed a decreased positivity in treated animals. Sirius red staining indicates a reduction in collagen content in animals receiving PR-PFD. (**B**) Quantification of collagen staining using Sirius Red in liver tissue (*p < 0.05). Arrows indicate inflammation nodules, lipid vacuoles, fibrosis and collagen bridges. (**C**)Percentage of alpha-SMA immunoreactivity in treated animals and controls, (**p < 0.01). Photos at ×20 magnification. *CVD* conventional diet, *HFD* high fat and high carbohydrate diet, *PFD* high fat and high carbohydrate diet + prolonged-release pirfenidone. Data are expressed as the media of the group ± SEM.

**Figure 3:**
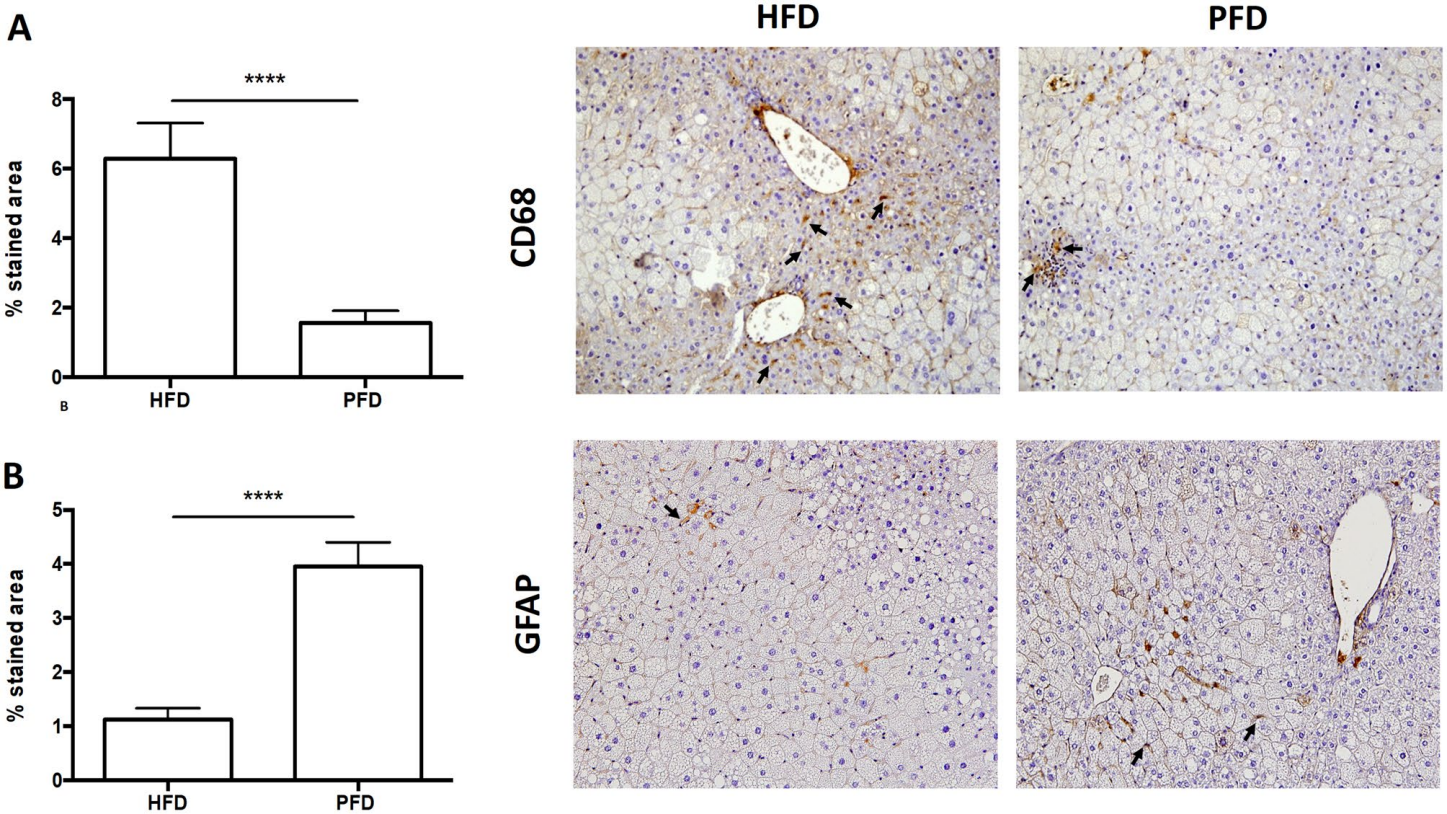
Immunoreactivity against CD68 and GFAP. (**A**) In animals fed with high fat-high carbohydrate diet an increase in CD68 immunoreactivity was observed. Animals treated with PFD showed a significant reduction in CD68 positive area (****p < 0.0001). (**B**) Quantification of GFAP positive area in liver tissue showed an increased in PFD animals (****p < 0.0001). Photographs at ×20 magnification. HFD: high fat and high carbohydrate diet and PFD: high fat and high carbohydrate diet + prolonged-release pirfenidone. Data are expressed as the media of the group ± SEM.

Histological analysis of liver of animals fed with HFD group showed significant tissue damage with steatosis, imminent fibrosis, and inflammatory changes predominantly in the periportal area with neutrophils and mononuclear cells. After prolonged-release pirfenidone treatment; as shown in H&E staining, a significant reduction in inflammation foci was achieved (p < 0.001) (Table 1, Fig. 2). To correlate this data, macrophage recruitment in the liver was evaluated using CD68 marker (Fig. 3A). Quantification of CD68 positive area was increased in HFD animals compared to PFD group. (6.29 ± 1.03vs. 1.57 ± 0.34, p < 0.0001). In particular, macrovesicular steatosis reached substantial reduction in PFD group compared to HFD mice group (Table 1, p < 0.0001); while microvesicular steatosis decrease in PFD group did not reached statistical significance (Fig. 2, Table 1). An imminent fibrosis development was observed, especially in periportal and pericentral areas. Fibrosis assessment was higher in HFD group; while histological improvement was observed in PFD animals (p < 0.01 and p < 0.05 for periportal and pericentral zones, respectively) (Fig. 2 Sirius red and Masson staining, Table 1). Likewise, a significant decrease in the number of fibrotic bridges was observed after administration of PFD versus HFD group (p < 0.05; Fig. 2 Masson staining, Table 1). To corroborate these facts, Sirius red collagen staining revealed lesser reactivity in PDF treated animals compared to non-treated HFD group (p < 0.05, Fig. 2B).

### Hepatic stellate cells activation in a MAFLD/NASH model was reversed in PFD treated animals

In order to monitor changes in HSC activation, immunohistochemistry for alpha-SMA was realized. As indicated in Fig. 2C, images were analyzed and percentage of area with immunoreactivity was calculated. HFD group showed a 2.28 ± 0.36% predominantly in the periportal area; whereas PFD group only had a 0.275 ± 0.055% (p < 0.01) indicating reduction of HSC activation in treated animals. This data also correlates with lesser fibrosis and collagen staining in this group. To evaluate if pirfenidone decrease α-SMA positive cells is due to reversal into the HSC quiescent phenotype, we performed GFAP immunohistochemistry (Fig. 3B). GFAP positive area was increased in PFD animals compared to HFD group. (3.96 ± 0.45 vs. 1.13 ± 0.2, p < 0.0001).

### Pro-inflammatory cytokines and Collagen 1 were downregulated

As expected, *Il1b* (301.1 ± 44.0 vs. 1285a ± 86.19, p < 0.0001), *Il6* (250.5 ± 181.9 vs 2692 ± 532.8, p < 0.01) and *Tnfa* (279.7 ± 5.774 vs. 683.0a ± 97.78, p < 0.05) mRNA levels were notably increased in HFD animals compared to CVD group. On the other hand, gene expression of these proinflammatory molecules were definitely diminished in PFD animals (565.3 ± 85.27, 1390 ± 477.3 and 220.6 ± 60.19 with p < 0.0001, p ≤ 0.05, p < 0.05; respectively) compared to HFD group. In addition, in Table 2*Col1a1* gene indicates that collagen expression is stimulated in a MAFLD/NASH model, but PFD administration diminish its mRNA levels (331.1 ± 64.63 vs. 97.45b ± 31.24, p < 0.01), This data correlated with previous reports of PFD effects in liver diseases^28^. Fold reduction is indicated in last column of Table 2 when comparing PFD vs HFD groups.

**Table 2:** Gene expression of proinflammatory cytokines and Collagen Type I.

Gene	CVD	HFD	PFD	Fold change versus HFD	Regulation
*Il1b*	301.1 ± 44.0	1285^a^ ± 86.19****	565.3^b^ ± 85.27****	− 2.27	Down
*Il6*	250.5 ± 181.9	2692^a^ ± 532.8**	1390^b^ ± 477.3*	− 1.94	Down
*Tnfa*	279.7 ± 5.774	683.0^a^ ± 97.78*	220.6^b^ ± 60.19*	− 3.09	Down
*Col1a1*	118.9 ± 38.03	331.1^a^ ± 64.63*	97.45^b^ ± 31.24**	− 3.40	Down

Data are expressed in mean ± SEM.

*CVD* conventional diet, *HFD* high fat and high carbohydrate diet, *PFD* high fat diet + prolonged release pirfenidone.

*p < 0.05, ****p < 0.0001 compared to CVD group and *p < 0.05, **p < 0.01, ****p < 0.0001 as compared with HFD group.

^a^Significance difference versus CVD group (*p < 0.05, **p < 0.01, ****p < 0.0001).

^b^Significance difference versus HFD group (*p ≤ 0.05, **p < 0.01, ****p < 0.0001).

### Microarrays analysis

**Figure 4:**
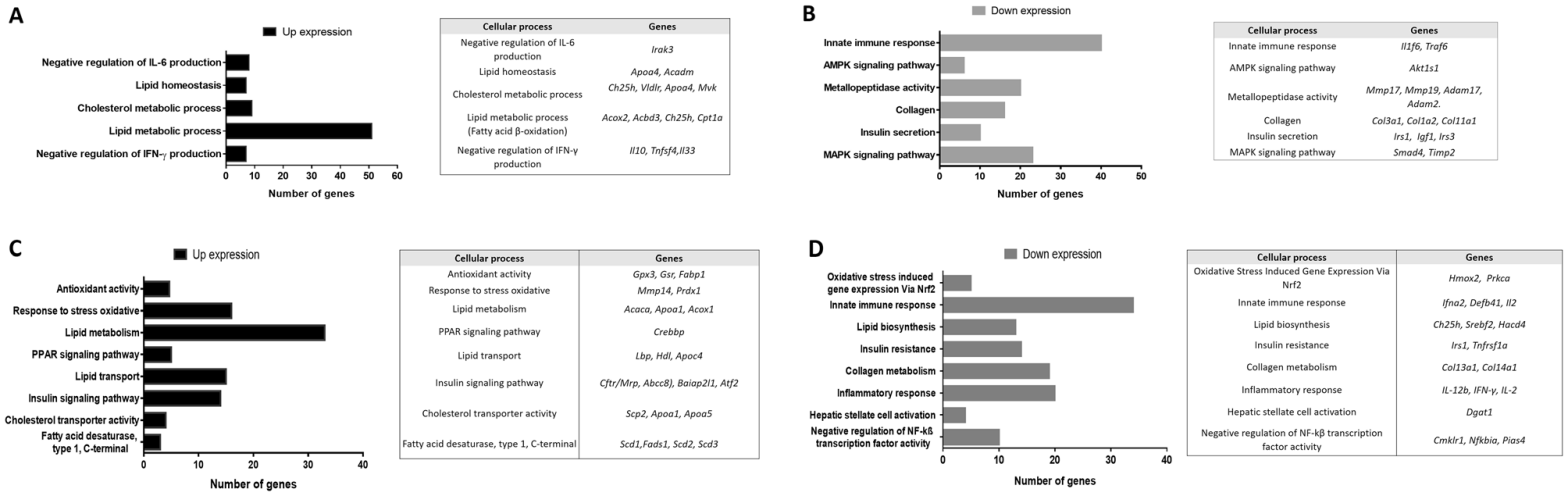
Microarray analysis of cellular pathways with gene dysregulation. CVD compared to HFD: (**A**) pathways with upregulated genes and (**B**) cellular tracks with decreased gene expression. PFD compared to HFD: (**C**) shows cell processes with gene overexpression and (**D**) metabolic pathways with decreased gene expression. *CVD* conventional diet, *HFD* high fat and high carbohydrate diet, *PFD* high fat and high carbohydrate diet + prolonged-release pirfenidone.

Double-channel chips were analyzed to compare 22,000 genes in mice genomes in the three experimental groups. As shown in Fig. 4A the comparison between CVD animals and HFD mice showed that 82 genes were overexpressed. Pathways associated were: lipid metabolic process (51 genes: *Acox2, Acbd3, Ch25h, Cpt1a*), cholesterol metabolic process (9 genes: *Ch25h*, *Vldlr*, *Apoa4, Mvk*), negative regulation of *IL6* production (7 genes: *Irak3*), lipid homeostasis (7 genes: *Apoa4, Acadm*) and negative regulation of IFN-gamma production (7 genes: *Il10*, *Tnfsf4*, *Il-33*). On the other hand, downregulated genes were those involved in: innate immune response (40 genes: *Il1f6*, *Traf6*), MAPK signaling pathways (23 genes: *Smad4*, *Timp2*), metallopeptidase activity (20 genes: *Mmp17*, *Mmp19*, *Adam17*, *Adam2*), collagen (16 genes: *Col3a1*, *Col1a2*, *Col11a1*), insulin secretion (10 genes: *Irs1*, *Igf1*, *Irs3*) and *Ampk* signaling pathway (6 genes: *Akt1s1*) as indicated in Fig. 4B. When comparing PFD group to HFD group 62 genes were upregulated, and main pathways involved are lipid metabolism (33 genes: *Acaca, Apoa1, Acox1*). Response to oxidative stress (16 genes*: Mmp14*, *Prdx1*), lipid transport (15 genes: *Lbp*, *Hdl*, *Apoc4*), insuling signaling pathway (14 genes: *Cftr/Mrp*, *Abcc8*), *Baiap2l1*, *Atf2*). *Ppar* signaling pathway (5 genes: *Crebbp*), antioxidant activity (5 genes: *Gpx3*, *Gsr*, *Fabp1*), cholesterol transport activity (4 genes: *Scp2, Apoa1, Apoa5*) and fatty acid desaturase, type 1, C-terminal (3 genes: *Scd1, Fads1, Scd2, Scd3*) (Fig. 4C). Genes which were downregulated are implicated in innate immune response (34 genes: *Ifna2, Defb41, Il2*), inflammatory response (20 genes: *Il12b, Ifng, Il2*). Also, downregulation was seen in collagen metabolism pathways (19 genes: *Col13a1, Col14a1*), insulin resistance (14 genes: *Irs1, Tnfrsf1a*), lipid biosynthesis (13 genes: *Ch25h*, *Srebf2*, *Hacd4*), negative regulation of *Nfkb* transcription factor activity (10 genes: *Cmklr1, Nfkbia, Pias4*), Finally, oxidative stress-induced gene expression via *NRF2* (5 genes: *Hmox2, Prkca*) and hepatic stellate cell activation (4 genes: *Dgat1*) (Fig. 4D) were suppressed as well.

## Discussion

Several studies have shown that specific miRNAs play a key role in the progression of metabolic associated fatty liver disease (MAFLD), mostly using high-fat diet animal models. Therefore, modulation of miRNA expression could be a potential therapeutic target for the treatment of this disease because miRNAs regulate lipid synthesis, glucose and fatty acid catabolism, inflammation, proliferation, apoptosis and necrosis; all processes epigenetically deregulated in NASH^15,23^. A comprehensive literature search was carried out to select some of the most representative miRNAs for each of the key processes found to be dysregulated in non-alcoholic steatohepatitis, which are inflammation, fibrosis, steatosis and insulin resistance. Therefore, alterations on miRNAs target genes involved in liver energy metabolism, inflammation, cell regeneration and fibrogenic signaling; driving the progression from MAFLD to NASH were considered. Our study is the first to show that miR-21a-5p, miR-122-5p, miR-34a-5p and miR-103-3p expression and numerous of their target genes like, *Srebf1*, *Tgfb1*, *Fasn* and *Cpt1a* are modified by pirfenidone treatment in a MAFLD/NASH model as part of an improvement in molecular, histopathological and biochemical parameters. miR-21a-5p has been extensively studied in liver diseases, as well as its main target genes. miR-21a-5p hepatic expression is increased in animal models and in patients with MAFLD/NASH^38–40^. Similarly, we found a significant increased expression of miR-21a-5p induced by high-fat diet in C57BL/6J mice. miR-21a-5p is consider a profibrogenic miRNA by its effect on Smad7 and also participates in the accumulation of liver lipids by interacting with various factors, such as sterol regulatory element binding transcription factor 1 (*Srebf1*), 3-hydroxy-3-methylglutaryl-coenzyme A reductase (*Hmgcr*) and protein binding to fatty acids 7 (*Fabp7*)^41–43^. We found that miR-21a-5p expression was decreased in PFD group, as well as, *Srebf*1 and *Tgfb1*. miR-21a-5p hepatic diminution has been associated with improved glucose tolerance and insulin sensitivity, in addition to prevent hepatic steatosis and fatty acid absorption^44^. *Tgfb* signaling is crucial for fibrogenesis as has been convincingly demonstrated in numerous studies^45,46^. In our study, *Tgfb1* decrease in PFD group associates with decreased expression of *Col1a1*. These results correlated with histological analysis displaying a reduction in the number of fibrotic bridges, periportal and pericentral fibrosis and percentage of collagen stained area in PFD group. We also found a faded alpha-SMA positive area, indicating a contracted activation of HSCs due to PFD treatment; which correlates with an increase in GFAP (Glial fibrillary acidic protein) quiescent marker in HSCs in these group. As stated by Zisser et al. and others, quiescent HSCs are characterized by the cytoplasmic storage of vitamin A and markers include, PPARg, GFAP, and BAMBI^47–49^. These observed effects are caused by PFD; a drug widely known for reducing fibrosis and collagen deposition in various organs and diseases, including MAFLD models^28,50,51^. Nuclear receptor peroxisome proliferation-activated receptor alpha (*Ppara*) is target gene of miR-21a-5p. A study has shown that miR-21a-5p is increased in NASH patients, resulting in diminished expression of *Ppara*^52^. In this way, miR-21a-5p contributes to cell injury, inflammation, and fibrosis, through its inhibition of the *PPARA* signaling pathway^23^. Also, Sandoval-Rodriguez et al. had previously reported that protein expression of *Ppara* is diminished in a mouse MAFLD/NASH model and restored to similar levels than CVD in animals treated with PFD^51^.

miR-122-5p is one of the most abundant miRNAs in the liver, it constitutes 70% of all hepatic miRNAs. It has been reported that miR-122-5p is overexpressed in liver tissue of C57BL/6 mice with MAFLD, and in HepG2 and Huh-7 cells exposed to FFA. These in vitro models present an excess of lipid accumulation and triglycerides secretion, decreased expression of *Sirt1* and genes related to lipogenesis^53^. Similarly, in the present study, we found that miR-122-5p expression was remarkably elevated in HFD mice as determined by qRT-PCR. Conversely, former studies have shown that miR-122-5p is decreased in MAFLD models induced by HFD. Also, reduced levels of miR-122-5p were identified in liver biopsies of patients with NASH, at the most severe stage of the MAFLD spectrum, compared to control group^39^. A possible explanation for these divergent results is the diverse animals’ strains and variety of conditions of MAFLD induction, as well as the different co-morbidities and severity in patients with MAFLD.

miR-122-5p has been reported to be expressed primarily in hepatocytes and to target multiple enzymes in lipid metabolism, including fatty acid synthase (*Fasn*), 3-hydroxy-3-methyl-glutarylcoenzyme A reductase (*Hmgcr*), and *Srebf1* and *Srebf2*^54^. We found that miR-122-5p reduced expression in PFD group correlates with *Fasn* mRNA reduced expression in microarray. *Fasn* catalyzes the last step in fatty acid biosynthesis and therefore it is an important determinant of maximal liver ability to generate fatty acids by novo lipogenesis^14^.

Recently, miR-34a-5p has been shown to be specifically modulated in liver diseases. Circulating miR-34a-5p levels are high in patients with MAFLD and in animal models of steatosis^55^. Similarly, our data showed a higher hepatic miR-34a-5p expression, while PFD administration attenuates its induction. Also, a decrease in the expression of miR-34a-5p and an increase in its target genes *Sirt1*, *Ppara*, and *Insig2* has been reported when green tea was used to protect against MAFLD development in a rodent model^56^. *Hnf4a* is a target gene of miR-34a-5p known to modulate the regulatory elements of promoters and enhancers of genes involved in the metabolism of cholesterol, fatty acids and glucose. In microarray analysis, our data showed increased *Hnf4a* expression by PFD treatment. Specifically in the liver, *Hnf4a* activates hepatic gluconeogenesis and regulates expression of several genes, including apolipoproteins^57^.

miR-103-3p has been shown to regulate insulin sensitivity and glucose homeostasis, previous studies have found that miR-103-3p expression was increased in the liver of patients with MAFLD; as well as, in vitro and in vivo experimental models of MAFLD/NASH^58–60^. In our study, miR-103-3p expression was also detected increased due to high-fat diet induction of NASH. Silencing miR-103-3p improved insulin resistance. In contrast, the gain of function of miR-103-3p in the liver was sufficient to induce insulin resistance^21^. Other studies confirmed that high expression of miR-103-3p led to insulin resistance by decreasing the expression of caveolin-1, which is a direct target of miR-103-3p and a critical regulator of insulin receptor^21,22^. These data correlate with the effects seen after PFD treatment, since ITT was improved and miR-103 expression was reduced. In accordance with the miRWalk database 2.0, *Srebf1* is a predicted miR-103-3p target gene. *Srebf1* is one of the main regulators of de novo lipogenesis in MAFLD, and *Srebf1* overexpression contributed predominantly to lipids accumulation^61^. In resveratrol-treated obese rats, a significant decreased expression of miR-103-3p was reported, as well as, a decline in *Srebf1* protein expression^60^. According to these facts, in PFD animals we observed the same pattern regarding these molecules. In agreement to the miRWalk database 2.0, carnitine palmitoyltransferase 1a (*Cpt1a*) is a predicted target gene for miRNA-103-3p. *Cpt1a* is a key enzyme involved in mitochondrial β-oxidation, catalyzes the transfer of acyl groups from fatty acyl-CoAs to carnitine in the mitochondrial membrane for the translocation into the intermembrane space^62,63^. *Cpt1a* expression is decreased in our MAFLD model induced by high-fat diet, while increased in PFD group.

Key cellular processes are found to be altered in the development of non-alcoholic steatohepatitis (NASH). Using microarray analyses, we examined genes involved in lipid metabolism, inflammation, oxidative stress, fibrosis, insulin resistance, and how these pathways or cellular metabolism were modified in NASH and after administration of prolonged-release pirfenidone.

When comparing PFD group to HFD group, we found a decreased expression in genes involved in hepatic stellate cells activation, collagen metabolic processes and transforming growth factor β receptor binding, events involved in fibrosis progression, which is coherent with the well-known antifibrotic effect of pirfenidone in diverse fibrotic diseases^27,28,64,65^.

Particularly in NASH related to MAFLD, recently Komiya et al. and Sandoval-Rodriguez et al. found that pirfenidone improves liver fibrosis in mice models^50,51^. Gutierrez-Cuevas et al. also found in a model of MAFLD induced by High fat diet that PFD showed beneficial effects in cardiac fibrosis^66^.

Insulin resistance is a characteristic feature of MAFLD that contributes to its pathogenesis. Under conditions of insulin resistance, abnormally high levels of insulin are required to metabolize glucose and inhibit hepatic glucose production effectively due to reduced insulin sensitivity in peripheral tissues. Insulin resistance stimulates the pancreas to increase insulin secretion from the portal vein, leading to higher levels of insulin in the liver than in the periphery. High concentrations of hepatic glucose and plasmatic insulin are recognized as biomarkers of hepatic insulin resistance. Elevated fasting glucose levels are due to hepatic insulin resistance, while elevated concentrations of free fatty acids occur^67^. Microarrays showed a decrease expression in genes that participate in insulin resistance and in insulin secretion in the PFD group compared to HF group, which indicates that hyperinsulinemia might be caused by a high fat diet.

Activation of innate response of the immune system during NASH occurs mainly due to excessive accumulation of lipids that leads to hepatocytes damage, activating an inflammatory response that worsens the progress of liver disease. Inflammation is characterized by *Tnfa*, *Il1b*, as well as reactive oxygen species production^68^. Our data indicates a decrease expression in genes involved in the innate response of the immune system in PFD mice versus HFD group. Extensive experimental and clinical data support a central role for macrophages in the development and progression of NASH. Liver-resident Kupffer cells initiate inflammation and help recruit blood-derived monocytes^69^. Macrophage polarization to M1 phenotype (proinflammatory) contribute to NASH progression^70^. Here, we found a significant reduction in immunoreactivity of CD68 marker in PFD treated animals. These data suggest that pirfenidone is able to reduce inflammatory response activation, correlating with the already reported anti-inflammatory properties of PFD^28,71^.

*Nfkb* is a transcription factor involved in innate and adaptive immune responses, as well as in a number of pathological processes, such as inflammation. Under normal conditions, *Nfkb* is sequestered in the cytoplasm and binds to IKB proteins, inhibiting the nuclear localization of *NFKB*. *NFKB* activation is normally moderate, whereas, under conditions of insulin resistance, its expression in the liver is greatly increased. *Nfkb* translocation to the nucleus leads to upregulation of target genes encoding inflammatory mediators, such as *Tnfa* and *Il6*^72^. We found a decrease expression of genes related to the inflammatory response in PFD mice compared to the HFP group, which could be linked to a decrease in the negative regulation of *Nfkb* activity.

We also found a decreased expression in genes that participate in the metabolic process of lipids and fatty acids, mainly of genes that participate in lipogenesis in PFD animals compared to HFD group. On the other hand, microarrays showed an increase in the expression of genes related with activity of the cholesterol transporter, the transport of lipids and type 1 fatty acid desaturase. Previous studies have reported changes in the expression of liver desaturases during the development of MAFLD/NASH, it was found that a significant decrease in the activity of *Fads1* in the progression to NASH^63^. Regarding cholesterol transport genes, hepatic accumulation of free cholesterol has been shown to be an outstanding feature in MAFLD, which correlates with the histological severity of the disease. Additionally, epidemiological studies have identified dietary cholesterol intake as a factor related to the risk and severity of MAFLD. Transport of lipids is important, since an excessive flow of free fatty acids into the liver can cause NASH. Diet is an important component in the development of MAFLD, since 15% of triglycerides in the liver have the diet as their source^73^. Fatty acids in the diet are involved in liver lipogenesis and could play a dual role in the pathogenesis of liver steatosis, since they are involved in its development and in its prevention or reversal depending of the amount of omega-fatty acids.

Lipotoxicity, arising from hepatic fat excess, leads to mitochondrial dysfunction associated with an elevated ability to oxidize fatty acids, resulting in the production of reactive oxygen species (ROS). In transcriptome analysis, a decreased expression in genes involved in the response to oxidative stress was present in PFD group compared to HFD group. MAFLD causes overexpression of antioxidant agents, but the unbalanced production of ROS promotes oxidative stress. Pirfenidone has been reported to have antioxidant capacity^74,75^ and this effect correlates with expected benefits in this MALFD model. Oxidative stress can induce hepatocellular damage by inhibiting enzymes involved in mitochondrial respiratory chain and inactivation of glyceraldehyde-3-phosphate dehydrogenase^76^. Furthermore, ROS cause lipid peroxidation and cytokine production, which contributes to hepatocellular injury and fibrosis, promoting the progression from simple steatosis to nonalcoholic steatohepatitis^68^. Oxidative stress in MAFLD induces hepatic stellate cell activation, the most important producer of extracellular matrix^77,78^. In summary, increased expression of miR-21a-5p, miR-34a-5p, miR-122-5p and miR-103-3p in this MAFLD/NASH model was reversed with prolonged-release pirfenidone. MAFLD/NASH group compared to conventional diet control revealed modifications in gene metabolic pathways implicated in lipid metabolic process, inflammatory response, antioxidant activity and insulin resistance; PR-PFD seems to reverse these modifications.

## Supplementary Information

Supplementary Information 1.
