# Supplementary material for: Pirfenidone modifies hepatic miRNAs expression in a model of MAFLD/NASH
Source: Sci Rep. 2021 Jun 3;11:11709. doi: 10.1038/s41598-021-91187-2 (PMC8175718; doi:10.1038/s41598-021-91187-2)
Supplement: Supplementary file 1 — Supplementary Information 1. [file 41598_2021_91187_MOESM1_ESM.docx]

**Pirfenidone modifies hepatic miRNAs expression in a model of** **MAFLD/NASH**

Escutia-Gutiérrez R, Rodríguez-Sanabria JS, Monraz-Méndez CA, García-Bañuelos J, Santos-García A, Sandoval-Rodríguez A and Armendáriz-Borunda J.

**Table 1**

Catalog number of specific probes used to evaluate the miRNA and gene expression.

| **miRNA** | **Catalog number** | **Gen** | **Catalog number** |
| --- | --- | --- | --- |
| mmu-miR-34a-5p | mmu481304_mir | *Tnfa* | Mm00443258_m1 |
| mmu-miR-21a-5p | mmu482709_mir | *II1b* | Mm00434228_m1 |
| mmu-miR-122-5p | mmu480899_mir | *Il6* | Mm00446190_m1 |
| mmu-miR-103-3p | mmu478253_mir | *Col1a1* | Mm00801666_g1 |
| mmu-miR-16-5p | mmu482960_mir | *Tgfb1* | Mm01178820_m1 |
|  |  | *Srebf1* | Mm00550338_m1 |
|  |  | *Mttp* | Mm00435015_m1 |
|  |  | *Cpt1a* | Mm01231183_m1 |
|  |  | *Fasn* | Mm00662319_m1 |
|  |  | *Gapdh* | Mm99999915_g1 |

*Tnfa* = tumor Necrosis Factor alpha, *II1b* = interleukin 1b, *Il6* = interleukin 6, *Col1a1=* collagen type I alpha 1 chain*,* *Tgfb1*= transforming growth factor beta 1*, Srebf1=* sterol regulatory element binding transcription factor 1, *Mttp=* microsomal triglyceride transfer protein, *Cpt1a* =carnitine palmitoyltransferase 1A, *Fasn=* fatty acid synthase*, Gapdh=* glyceraldehyde-3-phosphate dehydrogenase.
